# Influenza A Virus Challenge Models in Cynomolgus Macaques Using the Authentic Inhaled Aerosol and Intra-Nasal Routes of Infection

**DOI:** 10.1371/journal.pone.0157887

**Published:** 2016-06-16

**Authors:** Anthony C. Marriott, Mike Dennis, Jennifer A. Kane, Karen E. Gooch, Graham Hatch, Sally Sharpe, Claudia Prevosto, Gail Leeming, Elsa-Gayle Zekeng, Karl J. Staples, Graham Hall, Kathryn A. Ryan, Simon Bate, Nathifa Moyo, Catherine J. Whittaker, Bassam Hallis, Nigel J. Silman, Ajit Lalvani, Tom M. Wilkinson, Julian A. Hiscox, James P. Stewart, Miles W. Carroll

**Affiliations:** 1 National Infection Service, Public Health England, Porton Down, Wiltshire, United Kingdom; 2 Department of Infection Biology, Institute of Infection and Global Health, University of Liverpool, Liverpool, United Kingdom; 3 Clinical and Experimental Sciences, Faculty of Medicine, University of Southampton, Southampton, United Kingdom; 4 Department of Respiratory Infections, National Heart and Lung Institute, Imperial College, London, United Kingdom; German Primate Center, GERMANY

## Abstract

Non-human primates are the animals closest to humans for use in influenza A virus challenge studies, in terms of their phylogenetic relatedness, physiology and immune systems. Previous studies have shown that cynomolgus macaques (*Macaca fascicularis*) are permissive for infection with H1N1pdm influenza virus. These studies have typically used combined challenge routes, with the majority being intra-tracheal delivery, and high doses of virus (> 10^7^ infectious units). This paper describes the outcome of novel challenge routes (inhaled aerosol, intra-nasal instillation) and low to moderate doses (10^3^ to 10^6^ plaque forming units) of H1N1pdm virus in cynomolgus macaques. Evidence of virus replication and sero-conversion were detected in all four challenge groups, although the disease was sub-clinical. Intra-nasal challenge led to an infection confined to the nasal cavity. A low dose (10^3^ plaque forming units) did not lead to detectable infectious virus shedding, but a 1000-fold higher dose led to virus shedding in all intra-nasal challenged animals. In contrast, aerosol and intra-tracheal challenge routes led to infections throughout the respiratory tract, although shedding from the nasal cavity was less reproducible between animals compared to the high-dose intra-nasal challenge group. Intra-tracheal and aerosol challenges induced a transient lymphopaenia, similar to that observed in influenza-infected humans, and greater virus-specific cellular immune responses in the blood were observed in these groups in comparison to the intra-nasal challenge groups. Activation of lung macrophages and innate immune response genes was detected at days 5 to 7 post-challenge. The kinetics of infection, both virological and immunological, were broadly in line with human influenza A virus infections. These more authentic infection models will be valuable in the determination of anti-influenza efficacy of novel entities against less severe (and thus more common) influenza infections.

## Introduction

Influenza A virus (IAV) is an RNA virus of the orthomyxovirus family. IAV strains are endemic and largely asymptomatic in wild birds but certain strains can cause severe disease outbreaks in domestic poultry that are of economic importance. IAV strains also circulate in pigs, causing swine influenza. Seasonal influenza in humans is caused by IAV (and influenza B virus) strains that circulate in the global population and is managed by annual vaccination of those at high risk of serious disease (e.g. the elderly, those with chronic respiratory or cardiac disease, and pregnant women). Occasional zoonotic transmission of IAV and genome reassortment resulting in new strains of IAV cause pandemic outbreaks that are a worldwide health concern for persons of all ages [1]. The wildlife reservoir, antigenic evolution and rapid development of resistance to antiviral drugs mean there is a constant need to research and develop novel interventions. This relies on the availability of authentic animal models.

Animal models of human IAV infections include the ferret, guinea pig, non-human primate (NHP) and mouse, the last usually, but not always, requiring prior adaptation of human IAVs to cause disease [2–4]. The ferret is seen as the gold standard for pathogenicity and immunogenicity studies and has also been used extensively for transmission studies. However, cellular immune responses are not well characterised and there are few established reagents available for ferret T-cell immunology. There is therefore a need for a robust model of human IAV infection that will enable more authentic pathogenesis and protection/therapeutic studies.

The NHP model for influenza has the advantage of a well-characterised and human-like cellular immune response, as well as similar anatomy and physiology, because of the close phylogenetic relationship to humans. Studies on IAV in NHPs have often involved evaluation of highly pathogenic viruses, such as the zoonotic H5N1 viruses and 1918 pandemic H1N1 virus [2, 5–7]. Also, a number of published studies have looked at pathogenesis of the 2009 pandemic H1N1 virus (H1N1pdm) in macaques that involved high dose inoculation, typically ≥ 10^7^ plaque-forming units (pfu), by multiple routes, including the intra-tracheal (i.t.) route (for example: [8–11]). No published influenza NHP challenge studies since the 1940’s have examined solely the intra-nasal (i.n.) infection route, which is routinely used in human challenge studies [12–16].

Delivery of IAV by aerosol particles, which may be of more relevance to natural human infections, was reported in 1965–1974 for macaques [2]. One recent study delivered H1N1pdm virus to rhesus macaques by a combination of small-particle aerosol (10^6^ pfu) and direct bronchial spray (2x10^7^ pfu) [17]. However, IAV aerosol challenge in humans has not been used since the 1960’s, due to safety concerns [16, 18]. As well as concerns over safety, human volunteer studies have the disadvantage of much greater cost than non-human primate studies.

The importance of T-cell immunity as a correlate of protection for influenza has been highlighted in human studies in recent years [12, 19–21], and having an animal model which can recapitulate cellular correlates of protection is desirable.

The aim of this study was to define the parameters in NHP to model natural human infection, and hence a platform for pathogenesis, vaccine and therapeutics evaluation. We describe the pathogenesis and immune correlates using two novel IAV infection routes in cynomolgus macaques, namely i.n. droplets and small-particle aerosol and a clinically relevant human H1N1pdm challenge virus.

## Results

### Challenge with influenza A H1N1pdm does not result in clinical signs of infection

To establish an authentic model for human challenge by IAV, we chose to infect cynomolgus macaques (*Macaca fascicularis*) with A/California/04/09 (H1N1) (A/Cal/04/09). This virus has been extensively characterised in the ferret model by us and others [22], in which animals it usually produces a mild disease course representative of a typical non-complicated human H1N1pdm infection. In addition, previous studies have shown this strain is able to infect cynomolgus macaques [8, 10]. Finally, A/Cal/04/09 is genetically and antigenically very similar to A/California/07/09, which is still the prototype H1N1 strain recommended by WHO for the current (2015–16) seasonal vaccine.

Animals were challenged using one of three routes, intra-nasal (i.n.), intra-tracheal (i.t.) or inhaled aerosol (i.a.). Two doses, high (10^6^ pfu) and low (10^3^ pfu) were administered by the i.n. route. The i.t. dose was selected as 10^6^ pfu to allow direct comparison with the high-dose i.n. route. The i.a. dose (10^5^ pfu) was the maximum technically achievable for the A/Cal/04/09 stock virus. The routes and doses used in the 4 challenge groups are summarised in Table 1. None of the challenge routes used resulted in overt clinical disease in the NHPs, which was unsurprising given the reported lack of clinical signs observed in other studies using much higher challenge doses (>7x10^6^ pfu) of A/Cal/04/09 [8, 23]. Animal weight, temperature (rectal and by implanted chip), respiratory signs, red blood cell haemoglobin levels, C-reactive protein levels, and pre- and post-challenge thoracic x-rays were monitored, none of which showed changes attributable to infection (for example, weight changes are shown in S1 Fig).

**Table 1:** Challenge groups.

Group	Route of inoculation	Dose (log_10_ pfu per NHP)
**A**	Intra-nasal	6.0
**B**	Intra-nasal	3.0
**C**	Intra-tracheal	6.0
**D**	Inhaled aerosol	5.0

### Virus shedding following challenge

The effects of dose and route of infection on virus shedding were assessed in nasal wash, throat swab and broncho-alveolar lavage fluid (BALF). Virus shedding was evaluated either by plaque assay for infectious virus or by real-time PCR for genome load.

**Fig 1:**
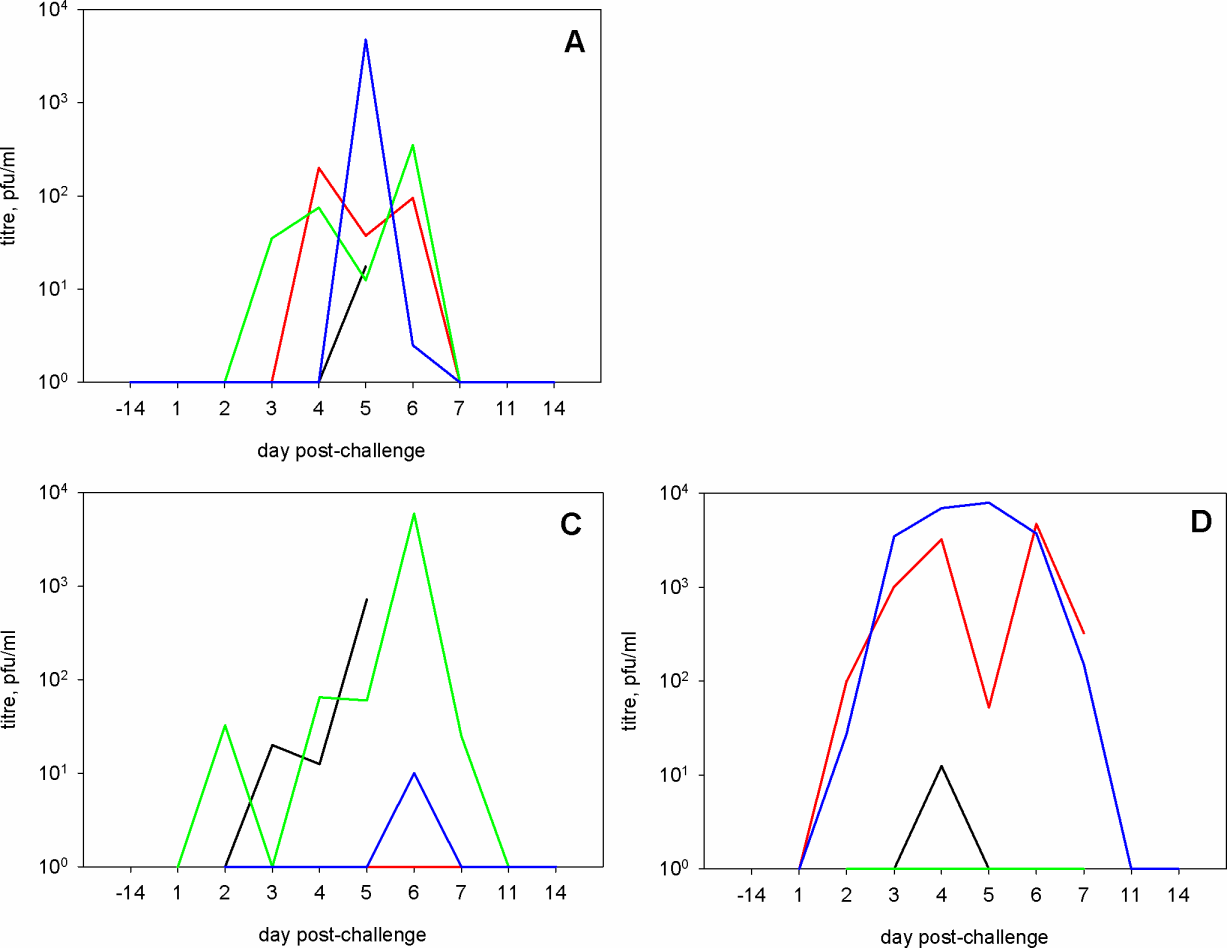
Virus infectivity in nasal wash fluid. Infectious virus was determined by plaque assay on MDCK cells. Titres are shown for individual animals. Upper panel: group A. For group B, all titres were below the limit of detection. Lower panel: groups C and D.

**Fig 2:**
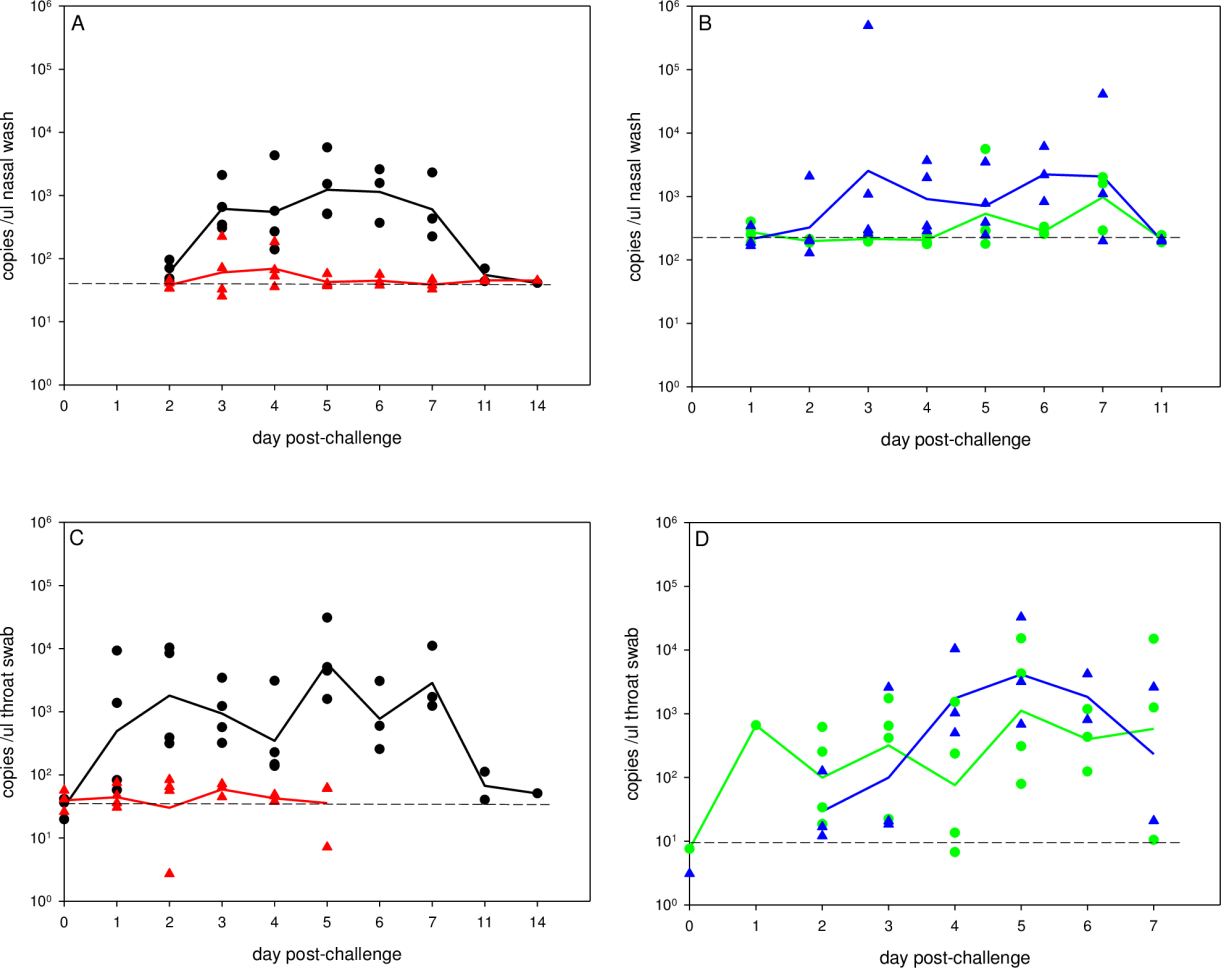
Viral RNA in nasal wash and throat swab fluid. RNA was extracted from fluids and viral load assessed by qRT-PCR for the M gene. Viral load is expressed as M gene copies per μl fluid. Panels A and B, nasal wash; panels C and D, throat swab. No throat swab samples were available for one of the NHPs in Group D. Panels A and C, i.n. challenge groups, high dose in black and low dose in red. Panels B and D, i.t. group in green and i.a. group in blue. In each panel, symbols represent loads for individual animals, solid lines show group means, and dashed horizontal line shows limit of detection.

In nasal wash fluid, infectious virus was shed by all four animals in group A (high dose i.n.), three of four animals in groups C (i.t.) and D (i.a.), but none of the group B (low dose i.n.) animals (Fig 1). Where shedding occurred, it was observed between days 2 and 7 post-infection (p.i.) and peaked between days 4 and 6 p.i. The time-course of viral RNA (vRNA) load in the nasal wash (Fig 2. panels A, B) followed that of infectious virus titres with vRNA not detectable after day 7 p.i. Most animals infected i.n. with a low dose did not show vRNA loads significantly above baseline at any time-point (Fig 2A).

In throat swabs, infectious virus was detected after i.t. and i.a. infection (S2 Fig) but not after i.n. challenge. The time-course of shedding of infectious virus in the throat broadly followed that of nasal wash, with virus undetectable by day 11 p.i. The time-course of vRNA load in the throat in i.t.- and i.a.-infected animals (Fig 2. panels C, D) followed a similar kinetic to that of infectious virus, with no vRNA detectable after day 7 p.i. However, real-time PCR was more sensitive than plaque assay as vRNA was observed between days 1 and 7 p.i. in high dose i.n.-infected animals (Fig 2C). Animals challenged i.n. with a low dose did not show vRNA loads significantly above baseline at any time-point (Fig 2C).

Virus shedding into the lumen of the lung was monitored by measuring vRNA in BALF collected at post-mortem (Table 2). Significant shedding was observed following i.t. and i.a. infection on days 5–7. Neither infectious virus nor vRNA were detected in BALF from animals challenged by the i.n. route.

**Table 2:** Viral RNA load in BALF, as M gene RNA copies /μl fluid.

	Days post-challenge			
Group	5	7	11	14
**A (high-dose i.n.)**	ND	-	-	-
**B (low-dose i.n.)**	-	-	-	-
**C (i.t.)**	++	(+)	-	-
**D (i.a.)**	+	(+)	-	-

Key: ++ > 10^4^; + 10^3^–10^4^; (+) 10^2^–10^3^;—< 10^2^ (background). ND, not done.

Thus, virus shedding was seen in all groups but following i.n. challenge virus detection was restricted to the upper airways and after low-dose i.n. delivery, virus was barely observed.

### Virus replication in NHP tissues following challenge

The viral load was assessed in respiratory tract tissues and lymph nodes of animals at multiple times p.i. Viral RNA loads in tissue homogenates were determined by qRT-PCR, relative to a synthetic A/Cal/04/09 M gene transcript of known concentration. The results (Table 3) show that i.n. challenge led to replication essentially confined to the nasal cavity (groups A and B). In contrast, vRNA was detectable throughout the respiratory tract in i.t. and i.a.-infected animals. A trend was observed for higher RNA levels, for a longer duration, in lungs following i.a. challenge (group D) compared to i.t. challenge (group C).

**Table 3:** Viral RNA loads in respiratory tract tissues.

	i.n. high dose				i.n. low dose				i.t.				i.a.			
Tissue	5	7	11	14	5	7	11	14	5	7	11	14	5	7	11	14
**NC**	++	+++	(+)	+	+	+	-	+	(+)	(+)	-	-	-	+++	(+)	(+)
**Tonsil**	-	(+)	-	-	-	-	-	-	+++	(+)	-	-	+	+	-	-
**Larynx**	-	-	-	-	-	-	-	-	(+)	-	-	(+)	(+)	-	-	(+)
**Trachea**	(+)	-	-	ND	-	-	-	-	(+)	-	-	-	-	(+)	-	-
**Bronchus**	-	-	-	-	-	-	-	-	+	-	-	-	+++	++	-	-
**U Lung**	-	-	-	-	-	-	-	-	(+)	++	-	-	+	(+)	+	-
**M Lung**	-	-	-	-	-	-	-	-	(+)	(+)	-	-	+	++	(+)	-
**L Lung**	-	-	-	-	-	-	-	-	+	(+)	-	-	++	+	-	-

Load is expressed as M gene RNA copies per mg tissue. Values of ≤ 10^3^ /mg were considered not significantly above background. +++ > 10^6^; ++10^5^–10^6^; + 10^4^–10^5^; (+) 10^3^–10^4^;—<10^3^ copies/mg. ND, not done. NC, nasal cavity; U upper; M middle; L lower lung lobe.

In lymph nodes (Table 4), low levels (10^3^−10^4^ copies/mg) of vRNA were detectable in numerous nodes from all the challenge groups. The exception was the hilar lymph nodes collected following i.t. challenge where a higher virus load was observed.

**Table 4:** Viral RNA loads in lymph nodes.

	i.n. high dose				i.n. low dose				i.t.				i.a.			
LN	5	7	11	14	5	7	11	14	5	7	11	14	5	7	11	14
**Sub.**	-	(+)	(+)	(+)	-	-	-	-	-	(+)	(+)	(+)	-	(+)	(+)	(+)
**Axil.**	-	-	-	(+)	(+)	(+)	-	-	+	-	-	-	-	-	(+)	(+)
**Hilar**	-	(+)	(+)	(+)	(+)	-	-	-	++	++	+	(+)	-	+	(+)	(+)
**Mesent.**	(+)	(+)	(+)	(+)	(+)	(+)	(+)	-	(+)	(+)	-	(+)	-	+	(+)	(+)

Load is expressed as M gene RNA copies per mg tissue. Values of ≤ 10^3^ /mg were considered not significantly above background. +++ > 10^6^; ++10^5^–10^6^; + 10^4^–10^5^; (+) 10^3^–10^4^;—<10^3^ copies/mg. LN, lymph node; Sub. Submandibular; Axil. Axillary; Mesent. Mesenteric.

Thus, like virus shedding, vRNA was observed in the tissues of all infected animals but was restricted after i.n. infection to the nasal cavity. Highest viral loads in the lung and bronchus were seen after i.a. infection.

### Histopathological effects of influenza challenge

To study the microscopic changes and potential cellular damage in NHPs after infection, samples from respiratory tract tissues, gastro-intestinal tract, spleen, tonsil, lymph nodes, liver, kidney, heart and brain, were examined using H&E-stained sections by a pathologist. Virus-induced lesions were not identified in tissues from outside the respiratory tract. Lesions observed in respiratory tissues were mild, and most could not be clearly attributed to virus infection, rather than the mechanical processes of necropsy and lung lavage. Neutrophil exudates were noted in the nasal cavity of one NHP (i.t. challenge, day 5 p.i.) as well as the cribriform plate of one NHP after high dose i.n. challenge (day 5 p.i.) and one NHP after low dose i.n. challenge (day 11).

**Fig 3:**
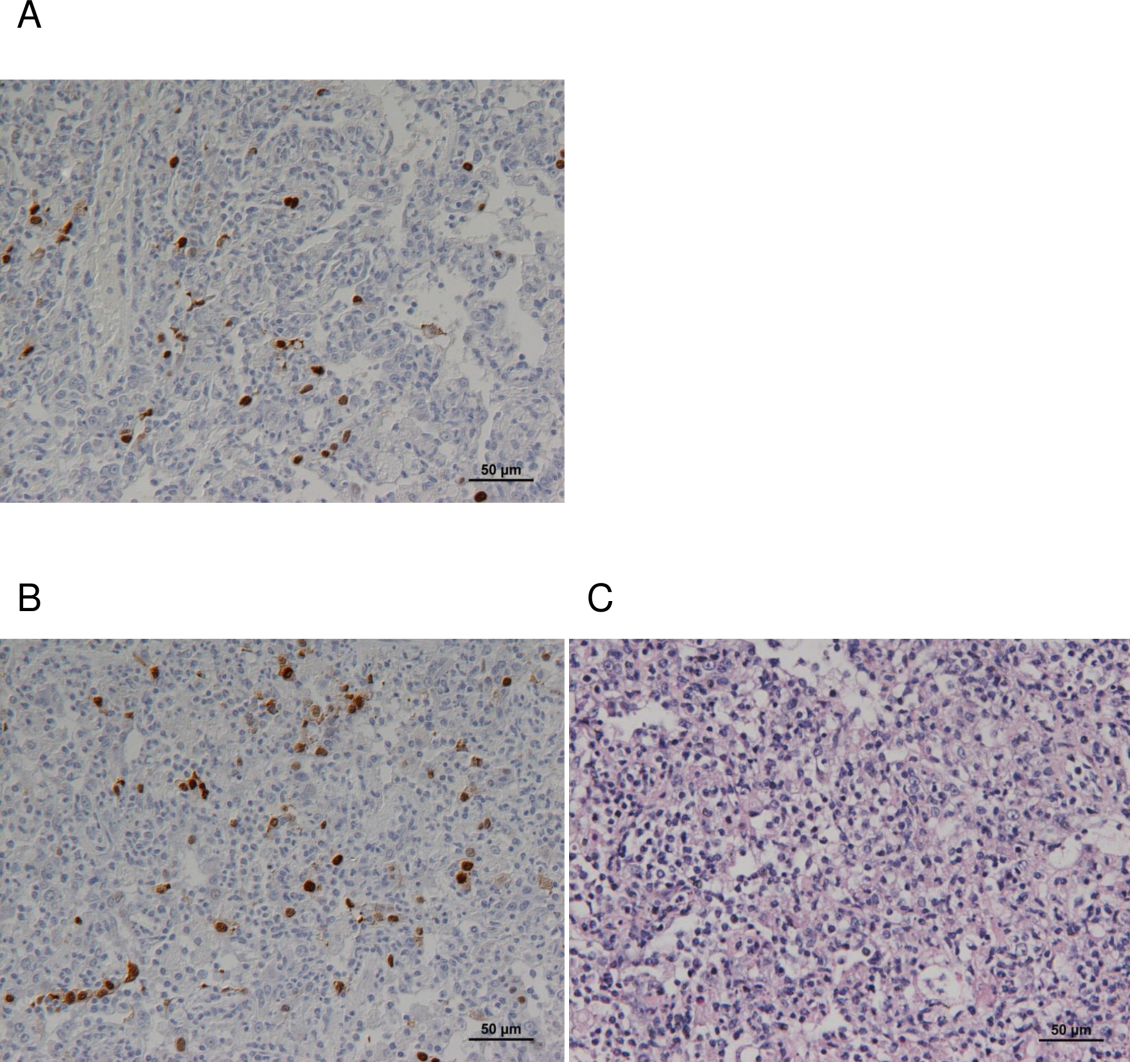
Detection of viral antigen in lung sections. Sections from (A) upper left and (B) lower left lung from one NHP (i.t. challenge 5 days post-infection) were stained with monoclonal antibody to IAV nucleoprotein and visualised by the PAP method (brown staining). Panel (C) shows an H&E stained section from the same sample as (B).

Subsequently, sections from the respiratory tract were stained for IAV NP antigen to visualise sites and cell types of virus replication. The IAV antigen was not detected in the majority of sections assessed. IAV NP staining was clearly detected in alveolar epithelial cells and macrophages within foci of lymphocytic inflammation in upper and lower lung lobes collected from one NHP 5 days after i.t. challenge (Fig 3).

### Animals from all challenge groups develop anti-IAV antibodies

**Fig 4:**
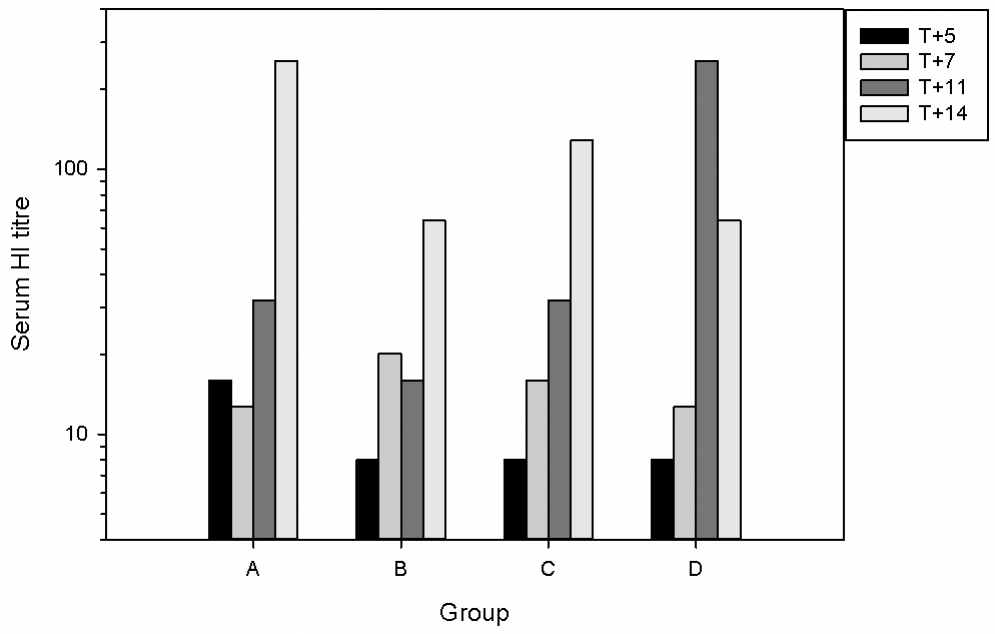
Serum HI titres against H1N1pdm virus. Serum was collected from all animals at necropsy, and from all remaining animals at 7 days post-challenge. Bars show Geometric Mean Titre (T+7), or titres from individual sera (all other days). Groups as in Table 1.

To determine the effect of challenge route on the development of antibodies to IAV, sera were collected at 5, 7, 11 and 14 days p.i. and the level of antibody assessed by haemagglutination inhibition (HI) analysis. The results (Fig 4) showed that animals in all groups sero-converted following challenge. HI titres generally increased over the time-course of infection in all groups. HI titres >40 (considered protective against IAV in human studies) were observed in animals sampled at day 14 of all 4 groups. A titre of >40 was reached most rapidly in the i.a. challenge group (by day 11).

### Effects of virus infection on immune cells in blood and BALF

To determine the cellular host response to IAV challenge, we analysed the quantity and phenotype of immune cells after challenge via different routes in various compartments including nasal wash, peripheral blood and BALF. We first analysed nasal wash fluid as, in the ferret model, i.n. challenge with IAV leads to a rapid 100-fold increase in immune cells [22, 24]. Viable cells in nasal wash fluids were counted, but there was no significant rise above baseline following challenge by any route (S3 Fig). This is in contrast to the innate cellular response to the virus seen in the nasal cavity of ferrets.

**Fig 5:**
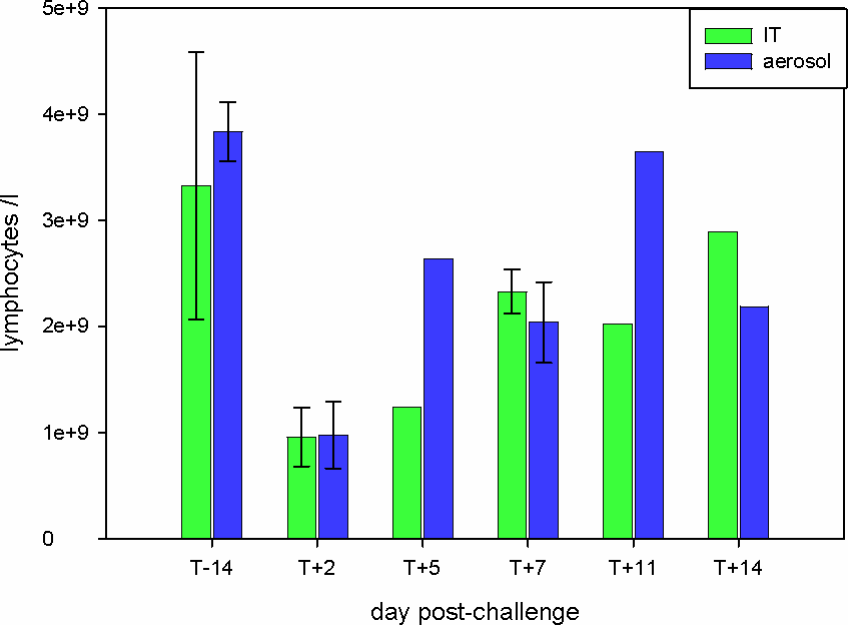
Lymphopaenia in i.t. and i.a. challenge groups. Whole blood was taken from animals and lymphocytes were counted using an IDEXX analyser. Results are expressed as cells/l. Group mean counts and standard deviation are shown.

**Fig 6:**
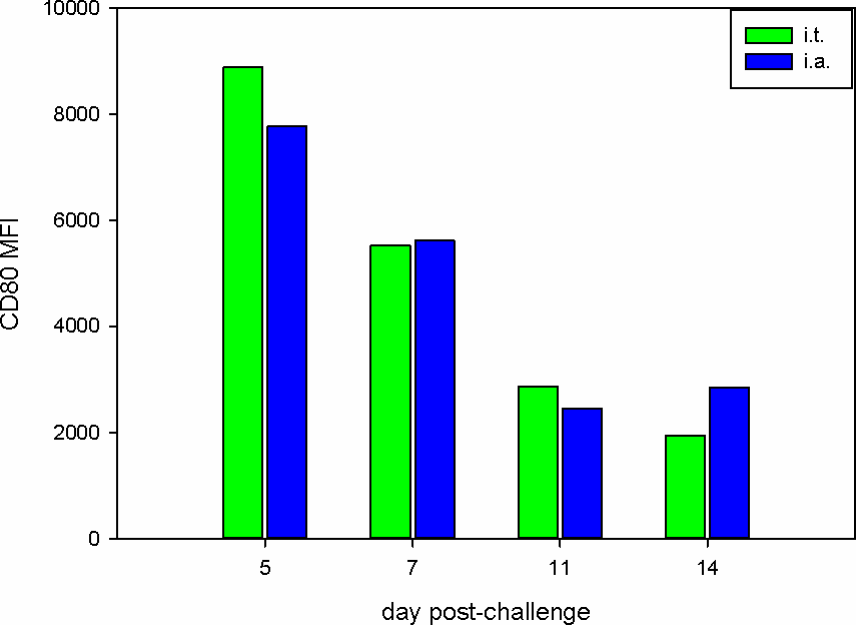
Viral challenge activates BAL macrophages. BAL cells from groups C (i.t.) and D (i.a.) were resuspended in FACS buffer and analysed for HLA-DR and CD80 expression by flow cytometry. Macrophages were defined as HLA-DR+ events and CD80 expression is presented as mean fluorescence intensity (MFI).

We next investigated the response to infection in blood. This analysis was limited to the i.t. and i.a.-challenged groups because suitable samples were not available for the i.n. groups. To determine the numerical and phenotypic changes, whole blood was analysed using light scattering flow cytometry. There were no significant changes observed in the number of red blood cells in any of the challenge groups. However, a marked lymphopenia was observed on day 2 post-challenge by the i.t. and i.a. routes (Fig 5). Lymphocyte counts were reduced 4-fold on day 2 (*p* < 0.02, 1-tailed t-test), then gradually returned to the pre-challenge values between days 5–14. This correlates with the lymphopenia observed in human influenza infections. In addition, lymphocyte:monocyte ratios in both i.a. and i.t. groups were all < 2.0 on day 2 post-challenge (S1 Table). This is another haematology marker observed in human influenza H1N1 infections [25]. Analysis of peripheral blood mononuclear cells (PBMCs) in i.t. and i.a.-infected groups by flow cytometry showed that there was little change in the percentage of CD4+ or CD8+ cells. The mean CD4:CD8 ratios were constantly around 5-6-fold at all time points p.i. The investigation of the response in BALF included determining the relative percentage of lymphocytes and macrophages and the activation status of macrophages. The majority of cells in these BALF samples from both these groups were macrophages, as determined by cytospin and HLA-DR-staining [26], at all time-points. By contrast, the percentage of both lymphocytes and neutrophils in the BALF peaked at day 7 in both groups (Table 5). Phenotypic analysis of macrophages was performed by FACS analysis for CD80 to measure macrophage activation. The results (Fig 6) showed a steady decline in the activation of macrophages (as defined by the intensity of CD80 expression), from a maximum at day 5 for both groups C (i.t.) and D (i.a.).

**Table 5:** Cytospin analysis of BAL cells.

	Inhaled aerosol			Intra-tracheal	
Day:	5	7	14	5	7
**Macrophages**	86.4	87.4	83.4	84.6	69.4
**Neutrophils**	1.0	7.4	1.8	3.4	11.6
**Eosinophils**	0.6	0.4	0.2	0.2	1.2
**Lymphocytes**	0.2	2.4	0.6	1.0	3.6
**Epithelial cells**	11.8	2.4	14.0	10.8	14.2

Fresh BAL cells were stained on cytospin slides, and 500 cells from each sample were phenotyped manually. Each cell type is shown as % of total cell population. The day 14 i.t. group sample was uncountable.

**Fig 7:**
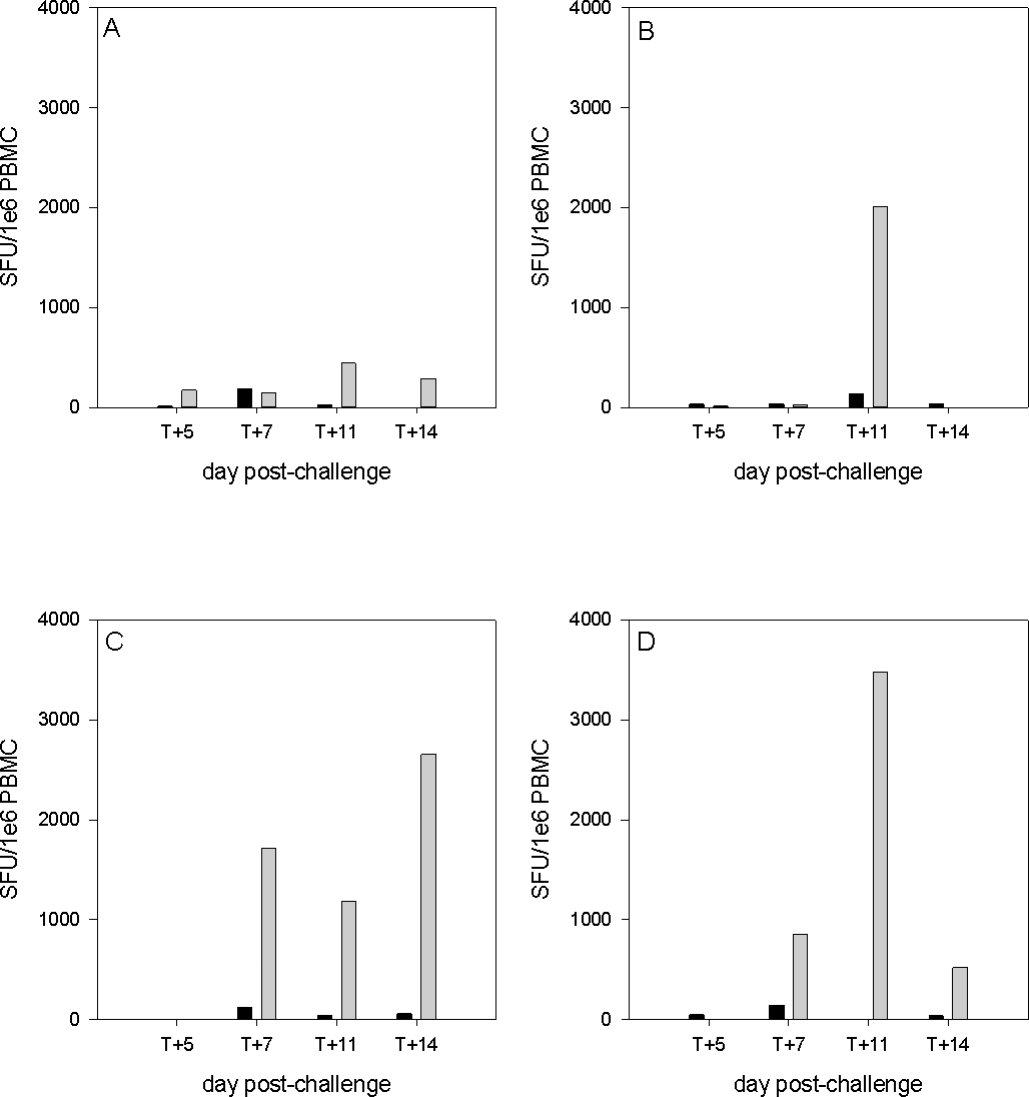
Interferon-γ secreting cells following stimulation of PBMCs with H1N1pdm virus. Each panel represents one of the challenge groups. For each NHP, the frequency of virus-specific cells measured by ELISPOT at day of cull (pale grey) and 4–6 weeks prior to challenge (black) are compared. Groups as in Table 1.

The frequency of influenza-specific IFNγ-secreting cells in peripheral blood was measured using an IFNγ ELISpot assay. Responses were detected in all four challenge groups before and after infection (Fig 7). However, apart from one individual animal, the responses in the i.n. challenged groups were extremely low. In both the i.t. and i.a. challenge groups, virus-specific IFNγ secretion was observed at 7, 11 and 14 days p.i., with the peak response seen at day 11 in the i.a. group and at day 14 in the i.t. group (3485 and 2657 spot-forming units (SFU)/ 10^6^ cells respectively).

Thus, while there were only small changes in the number and phenotype of immune cells in all groups, there was a good T cell response to the virus after i.t. and i.a.-challenge.

### Defining the proteome of BALF taken from naïve and infected NHPs

To investigate whether there was a difference in the BAL proteome from NHPs at different time-points post-infection, a label-free quantitative proteomic approach was used to identify and quantify proteins. BALF samples were obtained from 3 naïve NHPs as a control, and NHPs from group D (i.a. challenge) at days 5 and 7 post-infection. The data obtained from the mass spectrometry were analysed in three comparable sets. Analysis of the BAL proteome from a comparison between naïve NHP versus NHP at 5 days post-infection (panel A in S4 Fig) and naïve NHP versus NHP at 7 days post-infection (panel B in S4 Fig) identified and quantified a total of 509 proteins each. 49 proteins increased and 76 proteins decreased significantly in abundance in naïve versus 5 days post-infection. 67 proteins increased and 77 proteins decreased significantly in abundance in naïve versus 7 days post-infection. In a comparison between NHP at 5 days post-infection and at 7 days post infection, 721 proteins were identified and quantified, of which 58 proteins increased and 83 proteins decreased significantly in abundance (panel C in S4 Fig).

### Analysis of cellular proteins in BALF

**Fig 8:**
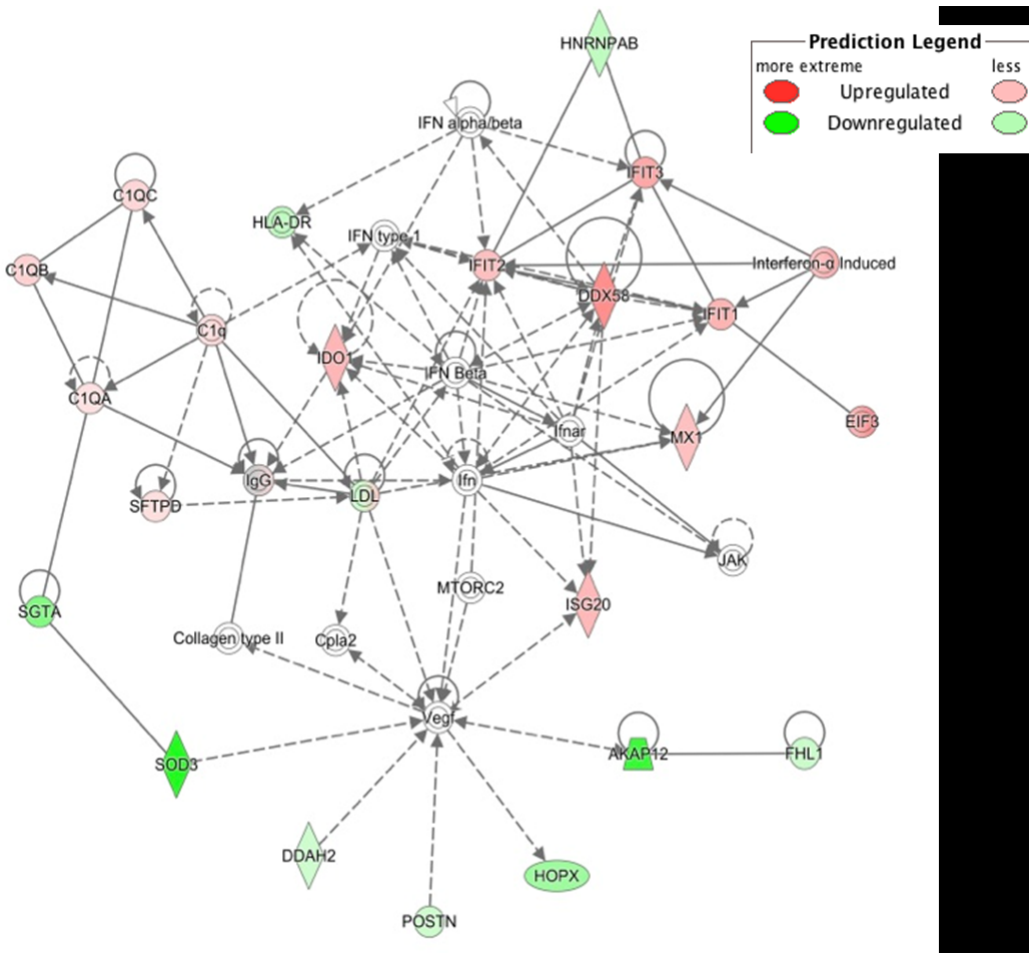
Network pathway analysis of proteins identified in the BAL fluids of samples from i.a. challenged NHP 7 days post-infection, compared to samples from i.a. challenged NHP 5 days post-infection. The network highlights proteins involved in immune response. Proteins in green highlight a 2-fold or more decrease in abundance in the NHP 7 days post-infection compared to samples from NHP 5 days post-infection. Proteins in red highlight a 2-fold or more increase in abundance. The shapes separate the different molecular classes. The solid lines represent a direct molecular interaction and the dashed lines an indirect molecular interaction.

The data indicated that at day 5 and day 7 post-infection proteins associated with the innate and adaptive immune responses had differential abundance when compared between these time points and to BALF taken from the naïve animals. At day 7 post-infection there was an increase in type I interferons including interferon α (IFNα) and its variants, compared to day 5 post-infection. These specific proteins were not identified by mass spectrometry (because of low abundance compared to other proteins) and therefore their presence is inferred due to the known activation of the highlighted proteins. The abundances of interferon induced gene products such as IFN-induced proteins with tetratricopeptide repeats (IFITs) 1, 2 and 3 (Fig 8 and panel C in S4 Fig) are significantly higher at day 7 compared to day 5 (p-values of 0.014, 0.0054 and 0.0049 respectively). IFN-inducible guanylate binding protein (GBP) 1 and 2 (also known to be regulated by interferon [27]) were also significantly different (as determined by Progenesis) between day 7 post-infection and day 5 post-infection (panel C in S4 Fig).

There was a significant increase at day 7 relative to day 5 post-infection in abundance of interleukins (IL) including IL1B, IL2, IL4, IL5, IL6, IL10 and IL13. In addition, CXCL7 also increased in abundance at day 7 compared to day 5 post-infection. Proteins associated with the activation of lymphocytes were identified at day 5 post-infection but not at day 7 post-infection.

## Discussion

The aim of this study was to define the parameters of IAV challenge in NHPs to model natural human infection, and hence develop a platform for vaccine and antiviral drug development. This report describes the outcome of influenza A H1N1pdm virus challenge of NHPs using three routes. Two of these are infrequently used routes, namely intra-nasal only, and aerosol only. Previous studies using IAV in macaques have tended to be modelling severe pulmonary disease, and as such, usually delivered all or most of the viral inoculum intra-tracheally (e.g. [8–11]). Recently, one prior study reported using i.n. challenge only, with a very high dose (10^7^ pfu) of H3N2 virus [28], and another study published after the commencement of our study, used an aerosol challenge [29], although the latter was in addition to a high dose intra-bronchial spray. Neither of these two studies looked at viral distribution, kinetics or pathogenesis. Studies using the i.n. route alone, in rhesus and cynomolgus macaques, were published in the 1940’s, and aerosol challenge studies were published in 1965–1974 (reviewed in [2]). These studies all resulted in a mild or unapparent disease.

Of two prior studies using A/Cal/04/09 in cynomolgus macaques, both used a multiple infection route including both i.t. and i.n. inocula [8, 10]. As a control challenge route, we used i.t., although with a lower dose than the two previously cited reports. In agreement with those studies, we were able to detect virus in both nasal washes and lung tissue in our i.t. challenge group. In contrast, our i.n.-only challenge led to an infection confined to the nasal cavity. No virus infectivity was recovered from the 10^3^ pfu i.n. challenge animals, but virus infectivity was recovered from all 4 animals in the 10^6^ pfu i.n. challenge group. This indicates that cynomolgus macaques are considerably less sensitive, at least to this virus strain, than are ferrets [22]. It should be noted that human challenge studies using H1N1 or H3N2 viruses typically use 10^3^–10^6^ TCID_50_ as i.n. challenge [12, 13], or > 10^5^ TCID_50_ [15, 16], and infection rates can be < 50% for H1N1 virus [12]. Optimal infectious doses reported in recent human H1N1pdm challenge studies were > 10^6^ TCID_50_ [30, 31]. This suggests a similar sensitivity to i.n. H1N1pdm challenge between human volunteers and our NHPs; however humans differ in that it is unlikely that adults have no prior exposure to IAV.

The kinetics of shedding of infectious virus from the nasal cavity, and peak infectious titres, in this study, were similar between the 10^6^ pfu i.n., 10^6^ pfu i.t. and 10^5^ pfu i.a. challenge groups (Fig 1). This is similar to the kinetics and peak titres shed by human volunteers infected with influenza A viruses [12, 14, 30–32]. This contrasts with the ferret model, in which infection with similar doses of the same strain of H1N1pdm virus by the i.n. route leads to peak titres on days 1–2 post-infection of >10^6^ pfu/ml [22].

The small-particle inhaled aerosol challenge infected both upper and lower respiratory tract, and showed more prolonged viral RNA replication than the i.t. challenge group, as well as greater virus shedding in nasal wash, despite using 10-fold less virus in the aerosol challenge. This suggests aerosol may be a more effective way of delivering virus to the lower RT than the traditional i.t. route, presumably due to the reduced particle size (0.5–1 μm compared to 30–100 μm, respectively). The i.a. route is also more authentic in terms of naturally-acquired human infection than the combined-i.t. route used elsewhere. It has been estimated that a person could inhale > 10^5^ influenza virus genome copies in 15 minutes in the vicinity of an infected patient [33], although only a fraction of these particles may be infectious. It would be of interest to determine the lower dose limit for successful infection of NHPs by the i.a. route.

The changes in immune cell populations in nasal fluid, BALF and peripheral blood after IAV infection were either inapparent or mild in all challenge groups. Although, there was evidence of macrophage activation in the BALF of animals challenged i.t. and i.a. (Fig 6). In spite of this, animals in all groups developed antibody responses, although the titres were only of a level that would be considered protective on day 14, except for the i.a.-challenged group (days 11 and 14) (Fig 4). Likewise, there was a good peripheral T cell response only in the i.t. and i.a. groups (Fig 7). The virus-specific IFN-γ response in these 2 groups was in line with a previous study which used a 9-fold higher i.t. challenge in rhesus macaques [9]. Virus-specific IFN-γ responses were also observed at 7 days post-infection in human challenge studies using an H3N2 virus and an H1N1 virus from 2007 [12, 15]. Analysis of the proteome of BAL fluids taken at different days post-infection in the cynomolgus macaques resembles protein changes that have been previously described in other experimental model systems, including the cynomolgus macaque model. At day 7 compared to day 5 post-infection, there was an increase in the type I interferon response, and both showed an increased response over the naïve samples. The innate immune response observed in this study aligns with previous published studies carried out in ferrets, mice and other non-human primates infected with IAV [34–37]. The host immune response to the viral infection plays a role in exacerbating the severity of the disease and the convergence of host responses with one another further affects the overall host response to viral infection.

Infection of the macaques in our study was essentially subclinical, which is in line with previous reports. Indeed infection of cynomolgus macaques with an H1N1pdm virus using 4x10^8^ TCID_50_, a much higher dose than used here, and delivered intra-bronchially, led to only mild symptoms in half of the animals [38]. One clinical observation well known in human influenza infections is a lymphopaenia, along with a reduced lymphocyte:monocyte ratio, which correlate with the peak of symptoms [39]. These clinical markers were observed in our aerosol and i.t. challenge groups, despite the lack of other observable signs of disease.

In our model, minimal pathological changes were observed in the respiratory tract, although the presence of viral RNA in these tissues confirmed spread of the virus, at least in the i.t. and i.a. challenge groups. Cynomolgus macaques express significantly more human-like (α-2,6) receptors in trachea and bronchus than rhesus macaques [40], facilitating infection with human influenza viruses, although the distribution of human-like receptors in macaques does differ from the distribution in the human respiratory tract [41, 42]. The H1N1pdm virus used in this study was propagated exclusively in MDCK cell culture, in order to retain the α-2,6 receptor specificity. We detected viral antigen in lung sections of only one NHP, despite detecting viral RNA in numerous tissue samples. This may be a sampling artefact, in that the fraction of an organ sampled by thin sections is much less than in the 30 mg sample homogenised for RNA extraction, so the probability of detecting virus, which is presumed to be highly focally distributed, is much lower. Immuno-staining many more sections from each organ may have resulted in a larger number of antigen detections.

A potential weakness of this study is the small number of animals used in each group. This was an initial study involving a minimal number of animals for ethical reasons. However the results show sufficient promise to inform the design of a larger, more focused study to demonstrate the various challenge strategies, in particular the clinical relevance of aerosol administration.

In conclusion, we describe novel intra-nasal and aerosol challenge models for IAV infection of macaques. These models differ in outcome, such that intra-nasal challenge leads to upper respiratory tract infection exclusively, whereas aerosol challenge leads to efficient distribution of virus throughout the respiratory tract. A number of factors compare favourably with influenza infection in humans, including the kinetics and magnitude of virus shedding, and transient lymphopenia early after infection. Thus our studies offer an attractive approach for analysing interventions that will affect upper respiratory or lower respiratory infection as an alternative to human studies.

## Methods

### Virus and cells

Madin Darby Canine Kidney (MDCK) cells were obtained from ECACC (Porton, UK) and cultured in DMEM containing Glutamax (Gibco, UK) and 10% v/v foetal bovine serum (FBS). Influenza A/California/04/09 (H1N1) was obtained originally from CDC (Atlanta, USA) and passaged 3 times in MDCK cells in our laboratory. The virus genome sequence was verified by dideoxy sequencing, and no changes were observed from the sequences deposited in GenBank. Virus titres were determined by plaque assay on MDCK cells under a 0.6% w/v agar overlay, followed by staining with crystal violet.

### Animals

Male cynomolgus macaques (*Macaca fascicularis*) 3–3.5 years old and of Mauritian genotype were sourced from an established UK breeding colony. The colony was demonstrated by regular screening to be free from Herpes B virus (*Herpesvirus simiae*), Simian T-cell Lymphotropic Virus (STLV), Simian retrovirus (SRV), Simian Immunodeficiency Virus (SIV), *Mycobacterium tuberculosis* and Salmonella sp. In addition animals allocated to this study were screened for the absence of serum antibodies against influenza H1N1pdm and H3N2 viruses. For 6 weeks prior to challenge, animals were monitored for health, and blood samples were taken at regular intervals from the femoral vein into serum separation tubes for serum separation and into heparin sodium for isolation of PBMCs. At each blood sampling point animals were weighed, temperature taken, superficial lymph nodes palpated, haemoglobin levels were measured using a Hemacue haemoglobinometer (Hemacue Ltd, Dronfield, UK) and nasal swabs and nasal washings were taken.

Based on assessment whilst in the breeding colony animals were housed in socially compatible groups of 4 in accordance with the Home Office (UK) Code of Practice for the Housing and Care of Animals used in Scientific Procedures (1989), (now updated to Code of Practice for Housing and Care of Animals Bred, Supplied or Used for Scientific Purposes, 2014) and the National Committee for Refinement, Reduction and Replacement (NC3Rs) Guidelines on Primate Accommodation, Care and Use, 2006. These groups were randomly allocated to each challenge regime. For all procedures animals were sedated by intramuscular injection with ketamine hydrochloride (Ketaset, 100mg/ml, Fort Dodge Animal Health Ltd, Southampton, UK) at a dose of 10mg/kg. None of the animals had been used previously for any experimental procedures. Groups A and B were challenged by intra-nasal droplet instillation of virus diluted in PBS, using a volume of 0.5 ml per nostril, while under light sedation. Group C was challenged with 4 ml per animal of virus diluted in serum-free DMEM, via an intra-tracheal catheter (MADgic Laryngo-tracheal Mucosal Atomization Device, LMA). Each subject was anaesthetised with an intramuscular injection of a combination of ketamine hydrochloride (10 mg/kg. Ketaset, 100mg/ml, Fort Dodge Animal Health Ltd, Southampton, UK and medetomidine hydrochloride (50 μg/kg Sedator, Eurovet Clinical Health, Bladel, The Netherlands), and placed in ventral recumbency. The vocal chords were visualised using a laryngoscope and were sprayed with 2% w/v lignocaine hydrochloride (Intubeaze, Dechra Veterinary Products, Shrewsbury, UK) prior to insertion of the pre-sterilised catheter.

Group D was challenged by small-particle aerosol delivered via the AeroMP Henderson apparatus using a 6-jet Collison nebulizer (Biaera Technologies, Hagerstown MD, USA). For this procedure animals were sedated with a combination of ketamine-acepromazine (ACP) and atropine (100 mg/ml ketamine [Ketaset; Fort Dodge Animal Health, Southampton, United Kingdom], 10 mg/ml ACP [Novartis], and 0.6 mg/ml atropine sulfate [Martindale Pharmaceuticals, Romford, United Kingdom]) in a ratio of 5:1:1. Target acquired volume was 4 L. Virus stock was diluted to 10^7^ pfu/ml in DMEM + 0.5% BSA for nebulization. Presented dose was calculated from back-titration of nebulizer and impinger liquids, and also from the known spray factor for A/Cal/04/09, and was determined to be 10^5^ pfu per animal.

On specified days after challenge, one animal from each group was anaesthetised for collection of blood for serum and isolation of PBMCs and then euthanised by intracardiac injection of a lethal dose (140mg/kg) of pentobarbotol sodium (Dollethal, Vetoquinol Ltd, UK, 200mg/ml). At necropsy tissues were collected for virology, RNA extraction and histopathology. In addition, blood samples were taken from all animals at day 2 post-challenge, and all surviving animals at day 7 post-challenge.

### Ethics statement

All procedures were approved by the Public Health England Ethical Review Committee, Porton Down, UK and authorised under UK Home Office project licence number 30/3083. All procedures were performed according to the UK Animals (Scientific procedures) Act 1986. The procedures conducted in this study are all specified in the project licence in a specific protocol section which describes each step and any adverse effects that might be encountered. Each study plan is reviewed and signed off by the project licence holder, the scientific leader and the manager of the facilities before being given a unique study number. The facility operates to GLP and this process is defined in an SOP agreed by the management of the Establishment and is subject to quality audit. All animals were housed in socially compatible groups in cages approximately 2.5m high by 4m long by 2m deep. These cages were constructed with high level observation balconies and with a floor of deep litter to allow foraging. Further enrichment was afforded by the provision of toys, swings, feeding puzzles and DVDs for visual stimulation. In addition to standard old world primate pellets further food was provided by a selection of vegetables and fruit.

### Clinical monitoring

Animals were observed twice daily by carers for clinical signs that included depression, withdrawal from the group, aggression, coat quality, food and water intake, changes in respiration rate, or cough/sneezing/nasal discharge. Weight and temperature (by rectal thermometer) were measured during examination under sedation. Chip temperature was measured from a Biotherm chip inserted into the outer right thigh, recorded once per day from day 7 pre-challenge to day 14 post-challenge, taken at the same time each day (morning). Inguinal and axillary lymph nodes were assessed under sedation/at necropsy and size estimated using a qualitative scoring system. Sizes of left and right nodes were estimated individually.

### qRT-PCR analysis of vRNA

Total RNA was extracted from tissues, and M gene was quantified by reverse transcriptase real-time PCR, as described previously [22]. Absolute RNA copy numbers were determined using a standard curve of synthetic M gene transcript, and normalised to the weight of tissue extracted. Viral RNA in fluids (nasal wash, throat swab, BAL) was extracted using the QIAamp Viral RNA kit (Qiagen).

### Serum antibody

Influenza H1N1 specific antibody was determined by HI assay using 0.5% v/v chicken red blood cells [43]. All serum samples were treated with receptor-destroying enzyme (RDE, Denka Seiken Co., Japan) at 37°C followed by heat-inactivation, prior to HI assay. All animals had titres of < 20 prior to challenge. Sero-conversion was defined as ≥ 4-fold rise in titre.

### Haematology

Haemoglobin was measured at each bleed under sedation using a HemoCue haemoglobinometer. For groups C and D only, blood (1 ml) was collected into EDTA tubes and was analysed by laser flow cytometry (IDEXX VetLab Lasercyte). C-reactive protein was measured in sera using the Monkey CRP ELISA kit (Life Diagnostics).

### Immunology

PBMCs were purified using Percoll gradients (Sigma Aldrich, Dorset, UK), then cryopreserved in FBS containing 10% v/v DMSO at 10^7^ cells/ml. Cells from BALF were analysed without prior freezing. Macrophages in BALF were analysed for HLA-DR and CD80 expression by flow cytometry (HLA-DR-APC-Cy7, BD Pharmingen 335831; CD80-PE-Cy7, BD Pharmingen 561135). HLA-DR has previously been used to analyse human macrophages, and other markers for macaque macrophages were not readily available [26, 44]. BALF cells were loaded onto a cytospin slide and stained with rapid Romanowsky stain for phenotyping [45]. 500 cells were phenotyped for each sample.

### Histopathology

Tissues were fixed in 10% neutral buffered formalin, processed to paraffin wax, cut into 5–6 μm sections, stained with haematoxylin and eosin (H&E) and examined by light microscopy. Immunohistochemistry (IH) was performed using the peroxidase anti-peroxidase (PAP) method as previously described [46, 47]. Primary antibody was mouse anti-IAV NP (H16-L10-4R5 (ATCC® HB-65™)).

### ELISPOT

PBMCs were defrosted into pre-warmed medium (R10) consisting of RPMI 1640 medium (Sigma-Aldrich, Dorset, United Kingdom) with the addition of L-glutamine (2 mM) (Sigma-Aldrich, Dorset, United Kingdom), penicillin (50 U/ml)-streptomycin (50 μg/ml) (Sigma-Aldrich, Dorset, United Kingdom), 25 mM HEPES buffer (Sigma-Aldrich, Dorset, United Kingdom), 0.05 mM 2-mercaptoethanol (Invitrogen, Paisley, United Kingdom), and 10% heat-inactivated foetal bovine serum (Sigma-Aldrich, Dorset, United Kingdom).

An IFN-γ ELISpot assay was used to determine the number of IFN-γ secreting influenza-specific T cells in PBMC using a NHP IFN-γ kit (MabTech, Nacka, Sweden). Cells were stimulated with live Influenza A/California/07/09 (H1N1) at a MOI of 3.5. This virus was egg grown, therefore egg allantoic fluid was used as a negative control. Plates (Merck Millipore, Watford, United Kingdom) were coated overnight at 4°C with 15 μg/ml of IFN-γ antibody. A total of 200,000 PBMC were plated per well in 50 μl R10 medium, with or without antigen in duplicate and incubated for 18 h. Phorbol-12-myristate (100 ng/ml; Sigma-Aldrich Dorset, United Kingdom) and ionomycin (1 μg/ml; Merck, Watford, United Kingdom) were used as a positive control. After culture, plates were washed and incubated for 2 h with biotinylated anti-IFN-γ. Spots were developed by the addition of streptavidin-alkaline phosphatase and 5-bromo-4-chloro-3-indolyl phosphate (BCIP)-Nitro Blue tetrazolium (NBT) substrate. Spot counts from the duplicate wells were averaged. Data were analysed by subtracting the mean number of spots in the cells and allantoic fluid control wells from the mean counts of spots in wells with cells and antigen.

### Sample preparation for mass spectrometry (MS)

Protein from BAL fluids, after spinning out and discarding cells, was digested with 1% (w/v) RapiGestTM (Waters MS Technologies, Manchester, UK) and analysed by LC MS/MS. Label free proteomics was used to analyse and compare protein output. Proteins were separated by reverse phase liquid chromatography and peptides analysed on a Q-Exactive mass spectrometer (Thermo Fisher Scientific).

### MS data analysis

Thermo RAW files were imported into Progenesis QI (version 2.0, Nonlinear Dynamics). Each sample was homogenized and analyzed in triplicate. All sample replicates were run time-aligned using default settings and an auto-selected run as a reference. The false discovery rates were set at below 1% using Mascot Percolator and the search results were imported back to Progenesis. Several data processing steps were used including the removal of proteins identified with low confidence; proteins identified by a single peptide in each sample replicate, the p-value was set at 0.05 and proteins with abundances differing by 2-fold or more were recorded as being differentially abundant. Proteins identified with 2 or more peptides in sample replicates were included. The mass spectrometry proteomics data have been deposited to the ProteomeXchange Consortium via the PRIDE partner repository with the dataset identifier PXD003803 and 10.6019/PXD003803.

### Bioinformatics Analysis–Protein Pathway Analysis

Networks linking differentially abundant proteins and their effects were generated through the use of QIAGEN’s Ingenuity Pathway Analysis (IPA, Qiagen Redwood City, www.qiagen.com/ingenuity) using data sets containing accession gene identifiers, corresponding maximum fold change values and P-values uploaded into the application. To select for genes whose expression was significantly differentially regulated, a cut off of 1.0 was set to identify these genes. The application contains a global molecular network onto which these genes were overlaid. These genes were connected based on proven studies supported by references in the literature or known canonical pathways present in the application knowledge base.

## Supporting Information

S1 Fig Changes in bodyweight of the 4 challenge groups. A (i.n. high dose), B (i.n. low dose), C (i.t.) and D (i.a.). Each line represents an individual animal. Weight is expressed as % of weight on day of challenge.
(PDF)

S2 Fig Virus infectivity in throat swabs. Infectious virus was determined by plaque assay on MDCK cells. A, i.t. group; B, i.a. group. Titres are shown for individual animals. No samples were available for one animal in the i.a. group.
(PDF)

S3 Fig Nasal wash cell counts. Points show group mean and standard deviation.
(PDF)

S4 Fig Volcano plots of proteins of increased and decreased abundance in the BAL fluid of samples from IAV infected NHPs. (A) 5 days post-infection compared to samples from naïve NHP; (B) 7 days post-infection compared to samples from naïve NHP; (C) 7 days post-infection compared to samples from 5 days post-infection. Vertical dashed lines indicate a cut-off of 2 fold change between comparison groups, while the horizontal dashed line indicates a p value of < 0.05 to define cohorts of polypeptides (pink shaded areas) with significantly increased (right hand side) or decreased (left hand side) abundance in NHP (A) 5 days post-infection, (B) and (C) 7 days post-infection. Proteins highlighted in red have crucial roles in the activation of the innate and adaptive immune response and host response to viral infection.
(PDF)

S1 Table Lymphocyte:monocyte ratios in whole blood of NHPs challenged by the i.t. and i.a. routes. Values < 2.0 are highlighted in bold.
(DOCX)
