# Supplementary figures and images for: Influenza A Virus Challenge Models in Cynomolgus Macaques Using the Authentic Inhaled Aerosol and Intra-Nasal Routes of Infection
Source: PLoS One. 2016 Jun 16;11(6):e0157887. doi: 10.1371/journal.pone.0157887 (PMC4911124; doi:10.1371/journal.pone.0157887)

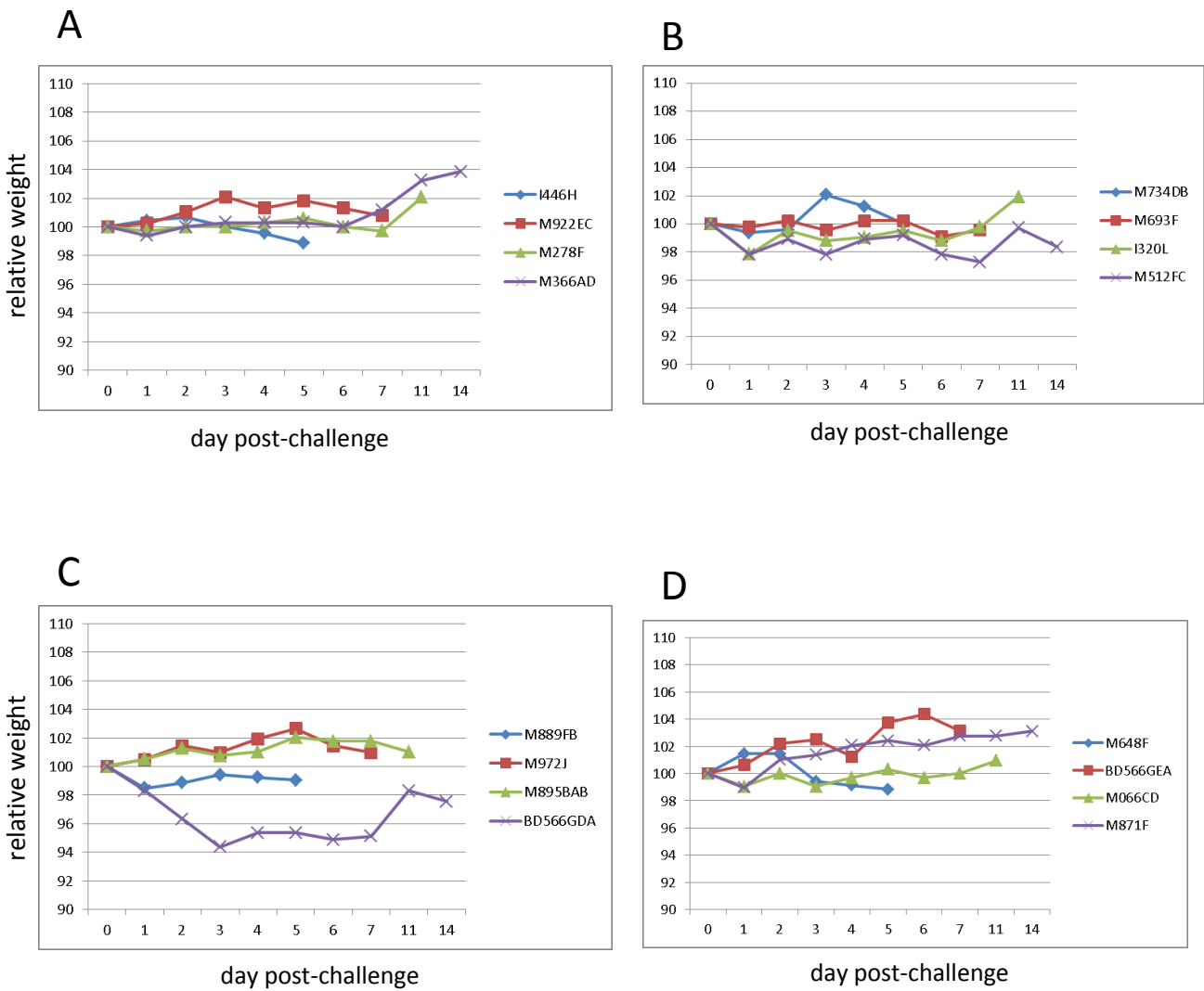

Supplement: S1 Fig — A (i.n. high dose), B (i.n. low dose), C (i.t.) and D (i.a.). Each line represents an individual animal. Weight is expressed as % of weight on day of challenge. (PDF) [file pone.0157887.s001.pdf]

S2 Fig.

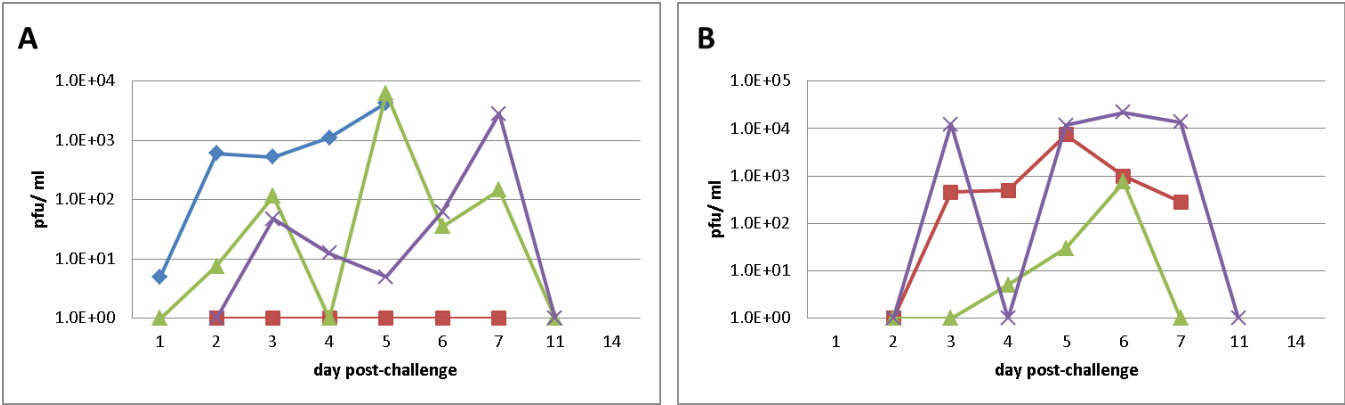

Supplement: S2 Fig — Infectious virus was determined by plaque assay on MDCK cells. A, i.t. group; B, i.a. group. Titres are shown for individual animals. No samples were available for one animal in the i.a. group. (PDF) [file pone.0157887.s002.pdf]

S3 Fig.

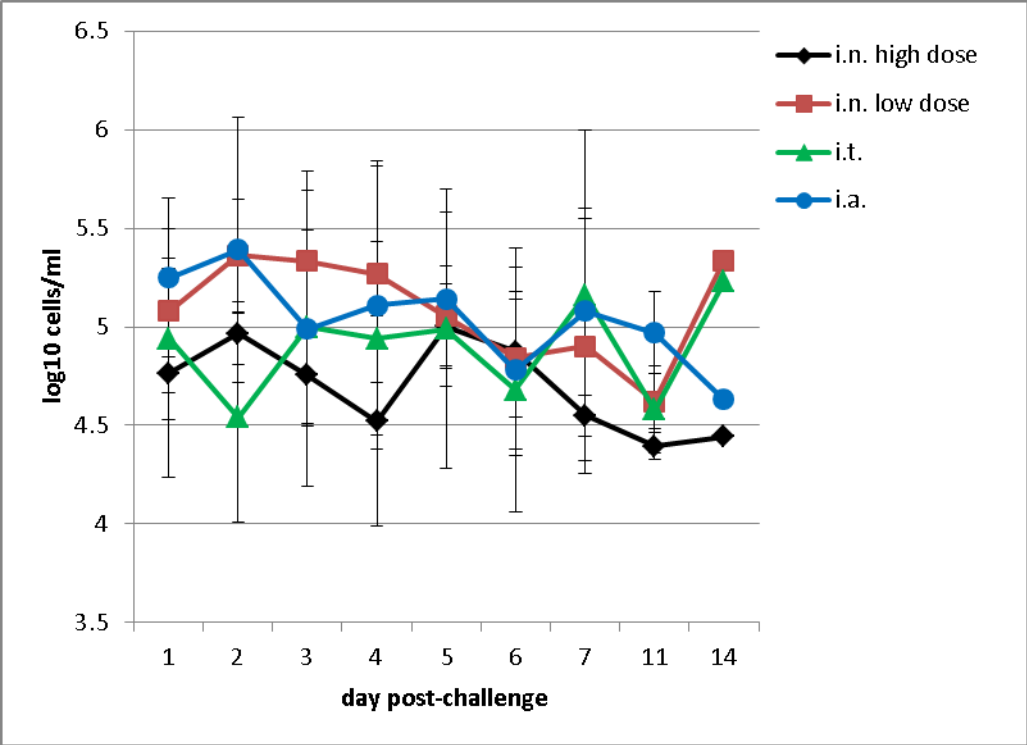

Supplement: S3 Fig — Points show group mean and standard deviation. (PDF) [file pone.0157887.s003.pdf]

A

Day 5 infected H1N1 NHP/naïve

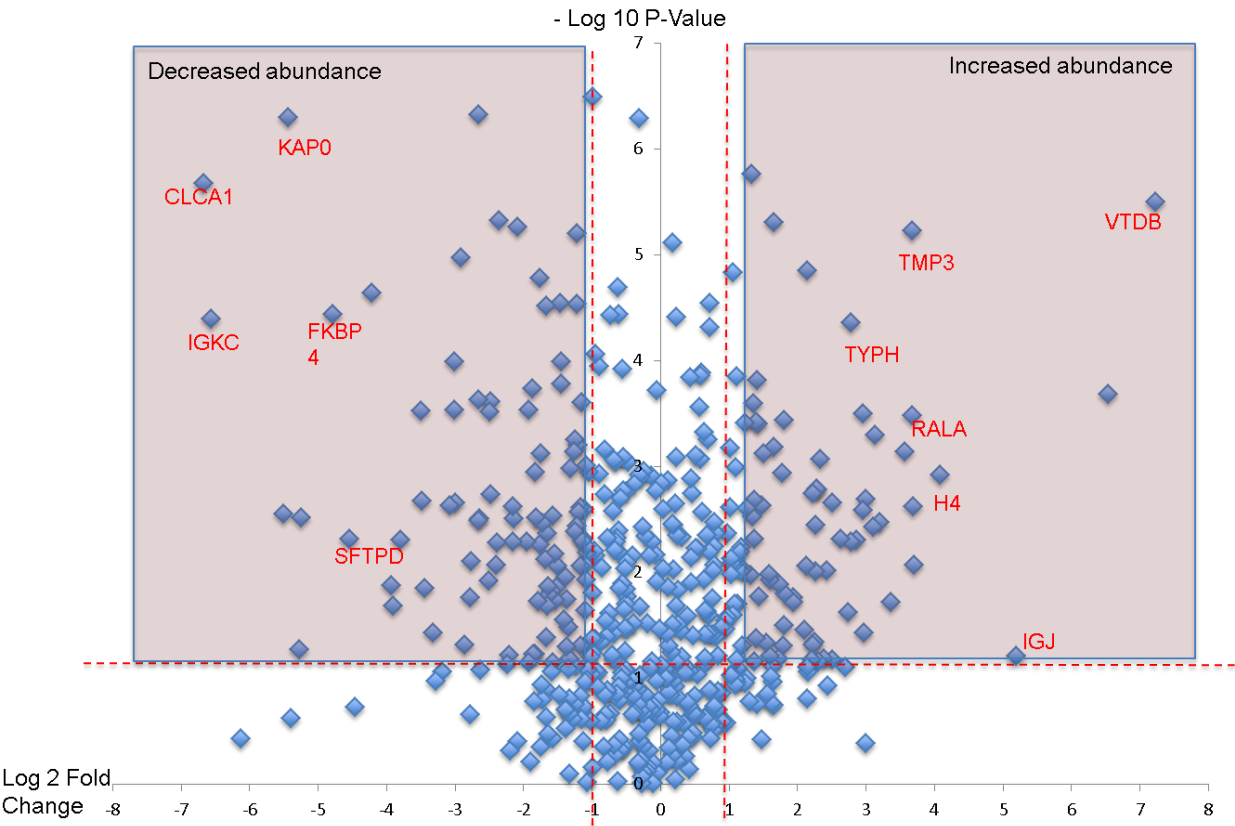

B

Day 7 infected H1N1 NHP/naïve

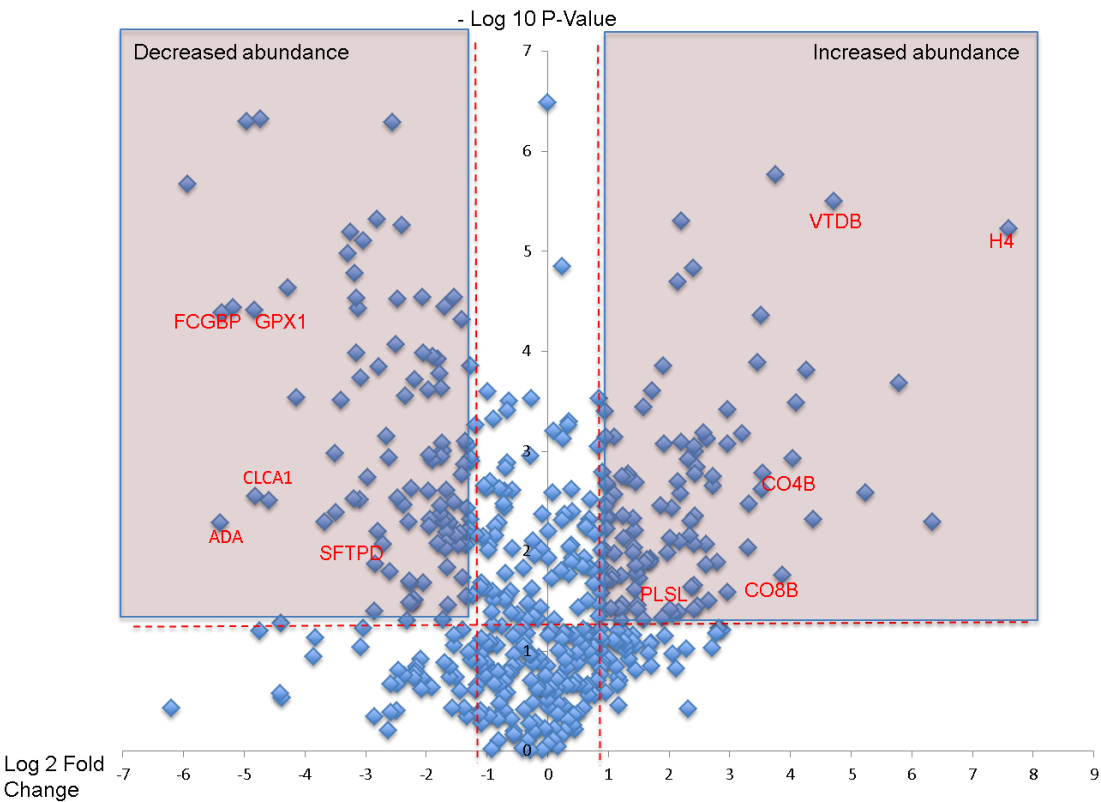

C

Day 7 Versus Day 5 H1N1 infected NHP Macaques

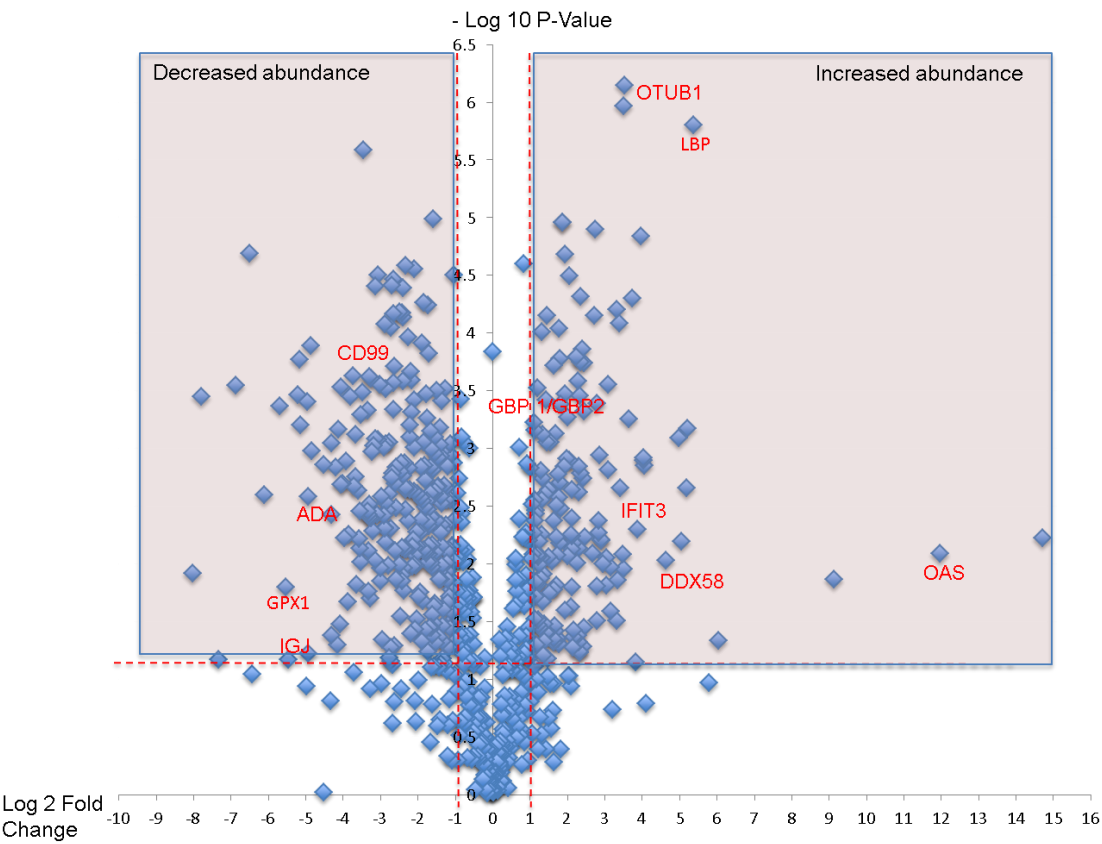

Supplement: S4 Fig — (A) 5 days post-infection compared to samples from naïve NHP; (B) 7 days post-infection compared to samples from naïve NHP; (C) 7 days post-infection compared to samples from 5 days post-infection. Vertical dashed lines indicate a cut-off of 2 fold change between comparison groups, while the horizontal dashed line indicates a p value of < 0.05 to define cohorts of polypeptides (pink shaded areas) with significantly increased (right hand side) or decreased (left hand side) abundance in NHP (A) 5 days post-infection, (B) and (C) 7 days post-infection. Proteins highlighted in red have crucial roles in the activation of the innate and adaptive immune response and host response to viral infection. (PDF) [file pone.0157887.s004.pdf]
