# Supplementary material for: Influenza A Virus Challenge Models in Cynomolgus Macaques Using the Authentic Inhaled Aerosol and Intra-Nasal Routes of Infection
Source: PLoS One. 2016 Jun 16;11(6):e0157887. doi: 10.1371/journal.pone.0157887 (PMC4911124; doi:10.1371/journal.pone.0157887)
Supplement: S1 Table — Values < 2.0 are highlighted in bold. (DOCX) [file pone.0157887.s005.docx]

**S1 Table.** **Lymphocyte:monocyte ratios in whole blood of NHPs challenged by the i.t. and i.a. routes.** Values < 2.0 are highlighted in bold.

|  | i.t. challenge | | | | i.a. challenge | | | |
| --- | --- | --- | --- | --- | --- | --- | --- | --- |
| Day post-challenge | *a* | *b* | *c* | *d* | *e* | *f* | *g* | *h* |
| -14 | 3.10 | 4.82 | 4.37 | 5.28 | 4.46 | 2.73 | 4.00 | 4.36 |
| +2 | **1.40** | **1.48** | **1.70** | **1.10** | **0.96** | **0.85** | **1.87** | **1.40** |
| +5 | **1.65** |  |  |  |  |  | 3.96 |  |
| +7 | † | 6.99 | 4.40 | 7.35 | 2.12 | 2.02 | † | 5.58 |
| +11 | † |  | † | 3.54 | 3.47 | † | † |  |
| +14 | † | 4.72 | † | † | † | † | † | 4.98 |

† animal culled prior to this time-point. Letters *a* to *h* refer to individual NHPs.
